# Unusually efficient photocurrent extraction in monolayer van der Waals heterostructure by tunnelling through discretized barriers

**DOI:** 10.1038/ncomms13278

**Published:** 2016-11-09

**Authors:** Woo Jong Yu, Quoc An Vu, Hyemin Oh, Hong Gi Nam, Hailong Zhou, Soonyoung Cha, Joo-Youn Kim, Alexandra Carvalho, Munseok Jeong, Hyunyong Choi, A. H. Castro Neto, Young Hee Lee, Xiangfeng Duan

**Affiliations:** 1 Department of Chemistry and Biochemistry, University of California, Los Angeles, California 90095, USA; 2 Department of Electronic and Electrical Engineering, Sungkyunkwan University, Suwon 16419, Republic of Korea; 3 Samsung-SKKU Graphene Center (SSGC), Suwon 16419, Republic of Korea; 4 Center for Integrated Nanostructure Physics, Institute for Basic Science (IBS), Suwon 16419, Republic of Korea; 5 Department of Energy Science, Sungkyunkwan University, Suwon 16419, Republic of Korea; 6 Department of Physics, Sungkyunkwan University, Suwon 16419, Republic of Korea; 7 School of Electrical and Electronic Engineering, Yonsei University, Seoul 120-749, Korea; 8 Centre for Advanced 2D Materials, National University of Singapore, 6 Science Drive 2, Singapore 117546, Singapore; 9 California Nanosystems Institute, University of California, Los Angeles, California 90095, USA

## Abstract

Two-dimensional layered transition-metal dichalcogenides have attracted considerable interest for their unique layer-number-dependent properties. In particular, vertical integration of these two-dimensional crystals to form van der Waals heterostructures can open up a new dimension for the design of functional electronic and optoelectronic devices. Here we report the layer-number-dependent photocurrent generation in graphene/MoS_2_/graphene heterostructures by creating a device with two distinct regions containing one-layer and seven-layer MoS_2_ to exclude other extrinsic factors. Photoresponse studies reveal that photoresponsivity in one-layer MoS_2_ is surprisingly higher than that in seven-layer MoS_2_ by seven times. Spectral-dependent studies further show that the internal quantum efficiency in one-layer MoS_2_ can reach a maximum of 65%, far higher than the 7% in seven-layer MoS_2_. Our theoretical modelling shows that asymmetric potential barriers in the top and bottom interfaces of the graphene/one-layer MoS_2_/graphene heterojunction enable asymmetric carrier tunnelling, to generate usually high photoresponsivity in one-layer MoS_2_ device.

## Abstract (web summary)

Vertical integration of two-dimensional materials can open unprecedented possibilities towards design of efficient optoelectronic devices. Here, the authors investigate the photoresponse properties of a graphene/MoS_2_/graphene heterostructure, revealing promising quantum efficiency performances.

## Graphical abstract

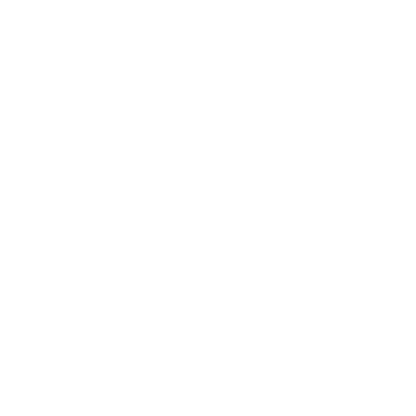

The two-dimensional (2D) layered materials such as graphene and semiconducting transition-metal dichalcogenides (TMDs) can exhibit peculiar electronic properties depending on their exact composition, thickness and geometry^1^,^2^,^3^,^4^,^5^. Furthermore, the broad possibility to combine different materials in van der Waals heterostructures (vdWHs) can create a new paradigm in materials science with unprecedented flexibility to integrate highly disparate materials and enable unique functions, as exemplified by the recent demonstration of vertical tunnelling transistors and vertical field-effect transistors for ultra-thin and flexible devices^6^,^7^,^8^,^9^,^10^,^11^,^12^,^13^ Graphene/multi-layer (ML)-TMD/graphene stack has also been shown to function as a unique photodiode for photocurrent generation or photodetection^7^,^12^. With a direct band gap in monolayer TMD and ultra-fast carrier transfer (∼1 ps) rate^14^, vdWHs with ultra-thin TMDs have attracted considerable interest for photovoltaic applications^15^,^16^ and ultra-fast photodetection^17^. However, classical charge transport theory for bulk semiconductors was often employed to interpret the electron transport in ultra-thin vdWHs even though it is well expected to exhibit completely different photocurrent generation characteristics^7^,^12^. Notably, an atomically thin graphene/WSe_2_/MoS_2_/graphene *p*–*n* heterojonction has been investigated for photocurrent generation. However, Shockly–Read–Hall recombination and Langevin recombination in the WSe_2_/MoS_2_ *p*–*n* junction could compromise the carrier extraction performance^13^. The unique characteristics of ultrathin vdWHs are insufficiently explored to date. In particular, a systematic investigation of layer-number-dependent studies is lacking due to complications from highly variable nature of the van der Waals interfaces and extrinsic factors in creating vdWH devices.

Here we report a layer-number-dependent photocurrent generation in graphene/MoS_2_/graphene vdWHs by creating a device with two distinct regions containing one-layer (1L) and seven-layer (7L)-MoS_2_ to exclude extrinsic device factors. Significantly, we discover a surprisingly higher photoresponsivity and internal quantum efficiency (IQE) by going from ML MoS_2_ device to a monolayer device. The discretized electrostatic potential barriers were introduced to interpret the photocarrier tunneLling and extracting in ultrathin vdWHs.

## Results

### Photocurrent generation in graphene /1L-MoS_2_/graphene stack

**Figure 1:**
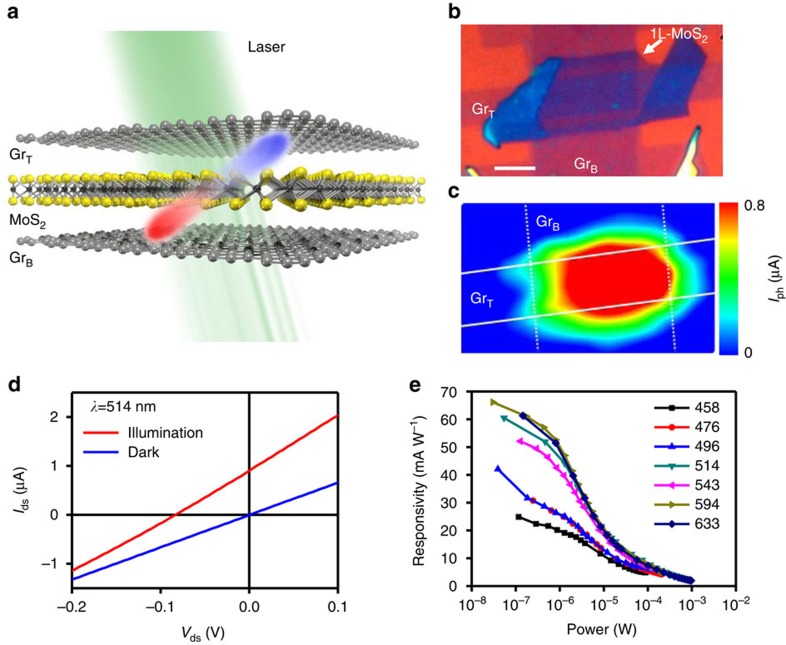
Photocurrent generation in the vertical heterostructure of graphene/monolayer MoS_2_/graphene stack. (**a**) A schematic illustration of the side view of the device, with a monolayer MoS_2_ sandwiched between the top (Gr_T_) and bottom (Gr_B_) graphene electrodes. Hole (red particle) and electron (blue particle) are generated in the monolayer MoS_2_ layer by 514 nm laser and transferred to top and bottom graphene electrodes. (**b**) Optical image of the vertical heterostructures with monolayer MoS_2_ sandwiched between the top (Gr_T_) and bottom (Gr_B_) graphene electrodes. The scale bar is 4 μm. (**c**) Scanning photocurrent image taken under a 514 nm laser with an excitation power of 400 μW and a spot size of 1 μm. The dotted and solid lines indicate the edge of the bottom and top graphene electrodes, respectively. (**d**) I-V characteristics of the device under laser illumination of 514 nm laser (red line) and dark (blue line). (**e**) Photoresponsivity of 1L-MoS_2_ heterostructures in various illuminated photon energies.

Figure 1 shows the schematic illustration of graphene/1L-MoS_2_/graphene heterostructure device on Si/SiO_2_ substrate. A focused laser was used to generate electron-hole pairs in the MoS_2_ layer, which can be separated by the asymmetric potential between the top graphene (Gr_T_)/MoS_2_ and the bottom graphene (Gr_B_)/MoS_2_ junction to produce photocurrent (Fig. 1a). Figure 1b shows an optical image of a typical graphene/1L-MoS_2_/graphene vdWH device. The 8 μm strip of Gr_B_ is located below 1L-MoS_2_ flake (vertical strip). The Gr_T_ layer is located directly on the MoS_2_ flake (horizontal strip) to overlap with MoS_2_ flake and the Gr_B_.The photocurrent generation in our devices was mapped by scanning photocurrent microscopy, where a focused laser beam was raster-scanned over the sample, while the photocurrent was being measured (Fig. 1c). The spatially resolved photocurrent map reveals pronounced photocurrent generation in the overlapping region in the vertical stack. The current–voltage (*I*_ds_–*V*_ds_) data obtained in the dark (blue line, Fig. 1d) and under 514 nm laser irradiation (red line, Fig. 1d) show a clear photoresponse in the vdWH. In contrast to typical diode characteristics observed in graphene/ML-MoS_2_/metal vdWHs due to asymmetric contact between top and bottom junction^7^,^12^, a linear transport curve is observed in the graphene/1L-MoS_2_/graphene heterostructure device, which can be attributed to direct tunnelling (*I*_DT_) through ultrathin tunnelling barrier of 1L-MoS_2_


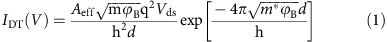


where *A*_eff_, *ϕ*_B_, *q*, *m*, *m*, *d* and *h* are effective contact area, barrier height, electron charge, free electron mass, effective electron mass, barrier width (MoS_2_ thickness) and Plank's constant, respectively^18^.

**Figure 2:**
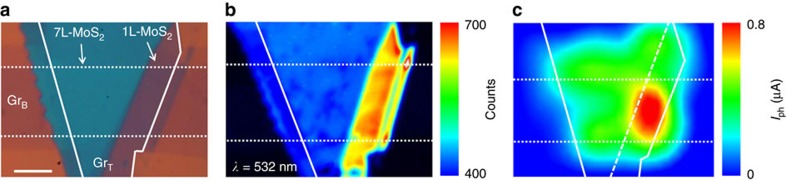
Comparison of photocurrent generation in monolayer and ML MoS_2_ heterostructures. (**a**) Optical image of the 1L MoS_2_ vertical heterostructures with partially 7L MoS_2_ sandwiched between the Gr_T_ and Gr_B_ electrodes. The dotted line and solid lines indicate the edges of the Gr_B_ and Gr_T_ electrodes, respectively. Scale bar, 5 μm. (**b**) The PL image of the same device. (**c**) Scanning photocurrent image of the same device taken under a 514 nm laser with an excitation power of 400 μW and a spot size of 1 μm. Dashed line indicates the boundary of 1L- and 7L-MoS_2_.

Based on the photocurrent response and input laser power, we can determine the photoresponsivity (A W^−1^) of the device. The photoresponsivity of 1L-MoS_2_ increases with decreasing the laser power could be attributed partly to absorption saturation in MoS_2_ and partly to the screening of a built-in electric field by the excited electrons in the conduction band of MoS_2_ (ref. 7). Importantly, the photoresponsivity of 1L-MoS_2_ increases with increasing the laser wavelength and a maximum photoresponsivity of 68 mA W^−1^ in 1L-MoS_2_ vdWHs was achieved at the wavelength of 633 nm and the laser power of 100 nW (Fig. 1e), which exceeds that of previous studies on graphene/ML-MoS_2_/graphene vdWHs device (∼30 mA W^−1^ without plasmonic enhancement)^7^,^12^. This performance difference is attributed to the number of layer difference between this work (monolayer MoS_2_) and previous reports (5∼30 nm MoS_2_), which will be further discussed by comparison of external quantum efficiency (EQE) and IQE between 1L-MoS_2_ and 7L-MoS_2_ vdWHs in Fig. 2g,h.

### Quantum efficiency between 1L-MoS_2_ and 7L-MoS_2_

To further unambiguously illustrate the difference between monolayer and ML MoS_2_ device, we have created a vdWH device with two distinct regions with 1L- and 7L-MoS_2_ in the same device (Fig. 2a). The 8 μm strip of Gr_B_ is located below the MoS_2_ flake (inside the dotted line) and the Gr_T_ layer is located directly above the MoS_2_ flake (inside the solid line), to overlap with MoS_2_ flake and the Gr_B_. The number of layers of MoS_2_ was then confirmed by Raman spectra measurements (Supplementary Fig. 1) and atomic force microscopic measurement. Figure 2b display the photoluminescence (PL) mapping image of 1L-MoS_2_ and 7L-MoS_2_ portion in Fig. 2a under an excitation wavelength of 532 nm (2.33 eV). The monolayer-MoS_2_ exhibited a much stronger PL compared with 7L-MoS_2_, which is consistent with the direct band gap nature of 1L-MoS_2_ (band gap ∼1.82 eV)^15^,^16^ and the indirect band gap nature of 7L-MoS_2_ (band gap ∼1.3 eV)^16^. Two prominent PL peaks can be identified at 630 nm (1.96 eV) and 680 nm (1.82 eV) in the spectrum (Supplementary Fig. 2), corresponding to A1 and B1 direct excitonic transitions^15^.

**Figure 3:**
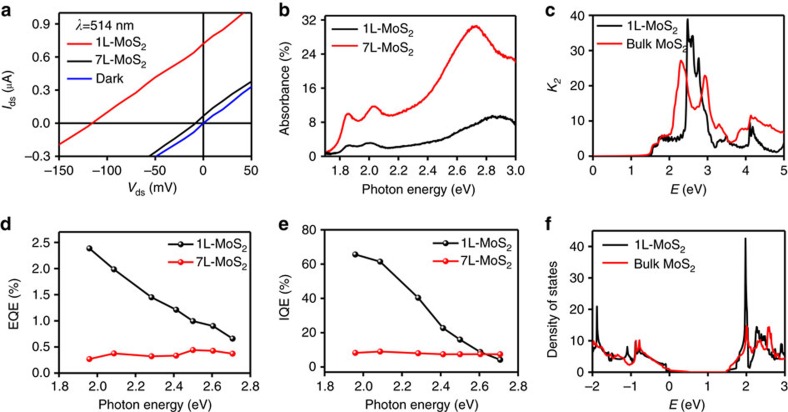
Quantum effieciency between monolayer and ML MoS_2_ heterostructures. (**a**) *I*_ds_–*V*_ds_ characteristics of the device under laser illumination of 514 nm laser (red line for 1L-MoS_2_, blue line for 7L-MoS_2_) and dark (black line). (**b**) Absorption spectra of a 7L-MoS_2_ flake (red line) and 1L-MoS_2_ flake (black line). (**c**) Calculated imaginary part of the dielectric function (*k*_2_) of 1L- and bulk MoS_2_ (as a model for thick ML). The effective thickness for the 1L-MoS_2_ was taken to be the inter-layer separation in ML-MoS_2_. (**d**) EQE of 1L-MoS_2_ stack and 7L-MoS_2_ stack at a laser power of 100 μW. (**e**) Photon energy dependence of IQE at a laser power of 100 μW (black line: 1L-MoS_2_; red line: 7L-MoS_2_). (**f**) Calculated density of states of 1L- and ML-MoS_2_.

The photocurrent map of the entire device clearly demonstrates that 1L-MoS_2_ region exhibited much more pronounced photocurrent than the 7L-MoS_2_ region (Fig. 2c). The current–voltage (*I*_ds_–*V*_ds_) data obtained in the dark (black line, Fig. 3a) and under 514 nm laser irradiation (red line for 1L-MoS_2_ region and blue line for 7L-MoS_2_ region; Fig. 3a) show a clear photoresponse in the vdWH. The open-circuit voltage and a short-circuit current obtained in the 1L-MoS_2_ region are 110 mV and 0.8 μA, both about one order of magnitude higher than those observed in the 7L-MoS_2_ (10 mV and 0.08 μA, respectively). This is a rather surprising and counter-intuitive discovery, considering that the 7L-MoS_2_ should have significantly higher optical absorption than 1L-MoS_2_ (Fig. 3b and Supplementary Note 1).

The enhanced photoresponsivity of 1L-MoS_2_ may be attributed to two potential factors: higher photocarrier generation rate in the monolayer MoS_2_ and higher photocarrier extraction/collection efficiency. We will discuss the influence of each factor below, first by modelling the transition probability and subsequently by comparing the internal and external quantum efficiencies. These two lines of reasoning will show that at peak IQE (633 nm and 1.96 eV), the second factor (that is, the improved collection) dominates in our device.

In general, the spectral dependence of the photocarrier generation rate can be estimated from first principles, using the Fermi golden rule. The probability of transition from a valence band state (υ) to a conduction band state (*c*) is thus given (in 2D) by


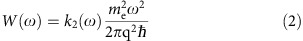


where *k*_2_ is the imaginary part of the dielectric function, *ħ* is the plank constant, *ω* is the frequency of the incident radiation, *m*_e_ and *q* are the electron mass and charge. In MoS_2_, *k*_2_ diverges because the conduction and valence bands run nearly parallel in some regions of the reciprocal space (that is, band nesting^19^). This can be noticed in Fig. 3c where we compare the imaginary part of the dielectric function, obtained from relativistic first-principles calculations, for 1L and thick multilayer (bulk) MoS_2_. Thus, *W* increases with the excitation energy up to the energy of the first peak, which is at ∼2.4 eV for the bulk and 2.8 eV for the monolayer, respectively^19^. The effect of band-nesting is more pronounced in monolayer, because this system is closer to the 2D limit and is responsible for a raise in the absorption coefficient in that spectral region.

To separate the effects of enhanced photon absorption from carrier collection, we have also conducted the photocurrent studies under difference excitation photon energies and determined the corresponding EQE (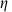, as defined by the number of carriers produced per incident photon^20^). The wavelength-dependent studies indicate the EQE of 1L-MoS_2_ increases with decreasing photon energy and can reach as high as 2.5% at 633 nm (1.96 eV; Fig. 3d), whereas the EQE of 7L-MoS_2_ maintains a relatively low value of 0.3–0.4% throughout the entire measured wavelength range (Fig. 3d). Thus, the increased EQE does not follow the trend of *k*_2_ that increases with increasing photon energy. This indicates that the enhanced absorbance is not the primary factor responsible for the maximum EQE. Other factors such as carrier collection should be considered.

Further insight is given by the IQE, which can be obtained by dividing the EQE by the optical absorbance of 7L- and 1L-MoS_2_ (Fig. 3e). Importantly, the maximum IQE in 1L-MoS_2_ can reach up to 65%, far exceeding to IQE in 7L-MoS_2_ (<7%). It is also noted that the IQE in 1L-MoS_2_ region steadily increases when the photon energy is reduced to approach the bandgap of 1L-MoS_2_ (1.82 eV (ref. 15)). The highest IQE reached ∼65% at 633 nm (1.96 eV), very close to the optical absorption edge of 1L-MoS_2_ (1.82 eV). It is noteworthy that in this region *k*_2_ is nearly independent on the thickness. According to the theoretical calculations, 1L-MoS_2_ has a much larger density of states near the band edges, at the valence band top due to the heavier hole masses, and near the conduction band edge, due to the presence of the 2D van-Hove singularities (Fig. 3f), increasing the probability of transfer to the graphene electrodes.

### Photocarrier extraction mechanism

Photocarrier transport in bulk MoS_2_ is driven by diffusion and drift process, which is dictated by different band bending at Gr_T_ and Gr_B_ MoS_2_ interface (originated from the difference of Gr_T_ and Gr_B_ doping densities)^7^,^12^ (Supplementary Fig. 3a). The photo-carrier extraction in atomically thin vdWHs is predicted to be completely different from that in bulk heterojunctions. Unlike bulk MoS_2_ having a continuous band bending (Supplementary Fig. 3a), atomically thin vdWH has discrete energy states in the vertical direction (Supplementary Fig. 3b). The classical semiconductor charge transport model—the charge drifts downhill following the band slope—cannot describe photocurrent generation phenomena via such discrete energy states in vdW heterojunctions. To this end, we introduce a tunnelling transport model to describe the photocarrier tunnelling through electrostatic potential barriers formed at the atomically thin vdW heterojunctions. Electrostatic potential barriers of 1L-MoS_2_ heterostructures are calculated by density functional theory (DFT; Supplementary Fig. 4 and Supplementary Note 2). Large electrostatic potential barriers are discretely constructed between Gr_T_/Gr_B_ and MoS_2_ interface. The barrier heights at the top and bottom junctions are symmetric because of symmetric environmental conditions imposed in the DFT calculations. In real device, the different environmental conditions of Gr_T_ and Gr_B_ result in asymmetric doping between Gr_T_ and Gr_B_. Doping characteristics of Gr_T_ and Gr_B_ were confirmed by 2D peak shift in Raman spectrum of another graphene/1L-MoS_2_/graphene heterostructure device (Supplementary Fig. 5 and Supplementary Note 3). With *n*- or *p*-type doping, the 2D Raman band exhibits a red or blue shift, respectively. In our device, the Gr_T_ is more *p*-type doped than Gr_B_. Such doping difference generates internal field, leading to asymmetric barrier height between top and bottom junctions (Supplementary Fig. 6). In this way, the photo-excited electrons can effectively tunnel to lower barrier at Gr_T_/MoS_2_ interface, whereas higher barrier at Gr_B_/MoS_2_ interface can block the electron tunnelling. Asymmetric tunnelling to top and bottom barrier thus generates photocurrent in vdWH device. It is noteworthy that the delta-function potential barriers for hole carriers are formed at the graphene/MoS_2_ interface, allowing hole tunnelling from MoS_2_ to Gr_B_ by built-in electric field^21^.

**Figure 4:**
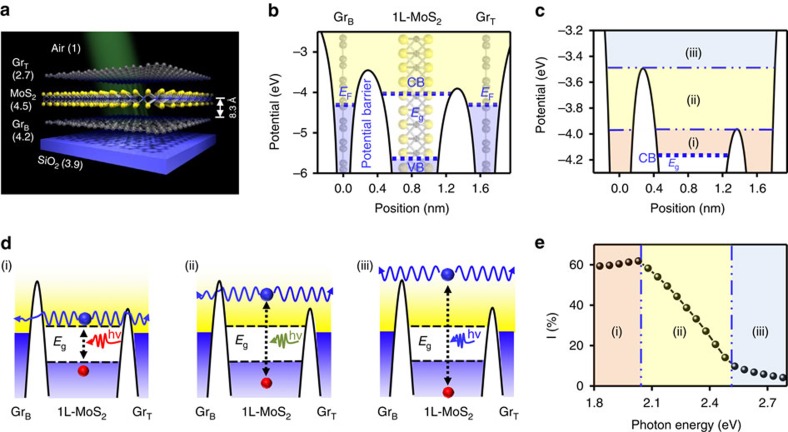
Electrostatic potentials of monolayer MoS_2_ heterostructure under different environments and photocarrier extraction mechanism. (**a**) Schematic images of graphene/1L-MoS_2_/graphene heterostructures with SiO_2_ substrate and air environment. Dielectric constant of each layers is indicated in brackets. (**b**) Electrostatic potentials of graphene/1L-MoS_2_/graphene heterostructures including environmental condition (Supplementary Note 3). Green coloured areas are the potential energy barriers. *E*_f_ is the Fermi level of graphene. CB, VB and *E*_g_ are conduction band, valence band and band gap of MoS_2_, respectively. (**c**) Electrostatic potentials near the conduction band of 1L-MoS_2_ heterostructures with three different potential barrier states. (**d**) Schematic images of photo-carrier tunnelling probabilities at the three different potential barrier states. (**e**) Calculated photocarrier extraction rate (*I=(T*_T_*–T*_B_)/(*T*_T_*+T*_B_)).

Furthermore, the different dielectric environment at the Gr_T_ and Gr_B_ /MoS_2_ interface can significantly alter the electrostatic potential. Figure 4a shows the schematic illustration of the dielectric environment of the device. The Gr_B_ is sandwiched between SiO_2_ substrate and 1L-MoS_2_, and the Gr_T_ between 1L-MoS_2_ and air. The dielectric constant of Gr_B_ (4.2) and Gr_T_ (2.7) are determined by the standard approximation of *ɛ*_GrB_=(*ɛ*_1L-MoS_2__+*ɛ*_SiO_2__)/2=4.2 and *ɛ*_GrT_=(*ɛ*_1L-MoS_2__+*ɛ*_air_)/2=2.7, respectively^22^,^23^, where *ɛ*_1L-MoS_2__=4.5 (ref. 24).

The calculated electrostatic potentials with these dielectric constants are shown in Fig. 4b,c (calculation is shown in Supplementary Note 4)^25^. Asymmetric electrostatic barriers are formed between the bottom (∼3.5 eV) and top (∼4.05 eV) junctions (Fig. 4b,c). The photocarrier extraction rate (*I*) can be determined by the difference of electron tunnelling probabilities between bottom and top junction barriers, which is normalized by the sum of tunnelling probability to top (*T*_T_) and bottom junction barrier (*T*_B_) (*I*)*=*(*T*_T_*–T*_B_)/(*T*_T_*+T*_B_)). The potential barrier was sliced to thin square barriers with width of 0.01 nm and then the tunnelling probability was calculated through overall square barriers (Supplementary Fig. 7).

Based on this model, the photocarrier extraction rate (*I*) strongly relies on the excitation photon energy (Fig. 4c,d). The tunnelling behaviour of excited electrons can be separated to three states, depending on the energy of excited electron, fully confined between *T*_T_ and *T*_B_, partially confined at the *T*_B_ and no confinement.

For fully confined between *T*_T_ and *T*_B_ (Fig. 4d–i), the excited electrons in MoS_2_ can be transported to Gr_T_ and Gr_B_ by tunnelling through both sides of the potential barriers. The tunnelling probabilities through *T*_T_ or *T*_B_ is defined to 
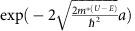
 (ref. 26), where *a* is energy barrier width, m^*^ is electron effective mass, *U* is electrostatic barrier, *E* is kinetic energy of electron and *h* is the Planck's constant. The top junction allows for higher tunnelling probability due to smaller barrier height compared with the bottom junction. Consequently, electrons can transfer to the Gr_T_ with positive *I*. The photocarrier extraction rate (*I*) is not altered appreciably in response to the energy of excited electrons (or absorbed photon energy), because effective barrier height (*U*–*E*) of the bottom and top junction changes by the similar magnitude (Fig. 4e–i).

For partially confined at the *T*_B_ (Fig. 4d–ii), the barrier at the bottom junction is higher than the kinetic energy of excited electrons. The tunnelling probability to *T*_B_ can be defined by 
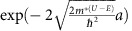
. On the other hand, the barrier at the top junction is lower than the energy of excited electron. The excited electrons transfer to Gr_T_ over the barrier with a tunnelling probability of 
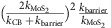
, where 
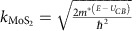
, 
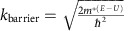
, where *U*_CB_ is conduction band minimum of MoS_2_ (refs 21, 26). The tunnelling probability through *T*_B_ increases with increase electron excitation energy (*E*) because of the decrease of effective barrier height (*U–E*), whereas the tunnelling probability to the *T*_T_ remains almost at 1 regardless of the electron energy. Consequently, the photocarrier extraction rate (*I*) decreases in proportion to electron excitation energy (Fig. 4e–ii).

For no confiment case (Fig. 4d–iii), both *T*_B_ and *T*_T_ heights are lower than the energy of excited electrons; therefore, the tunnelling probability to *T*_T_ and *T*_B_ are defined by 
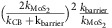
. The photocarrier extraction rate (*I*) decreases in proportion to the electron excitation energy (or absorbed photon energy; Fig. 4e–iii).

### MoS_2_ layer-dependent electrostatic potentials

**Figure 5:**
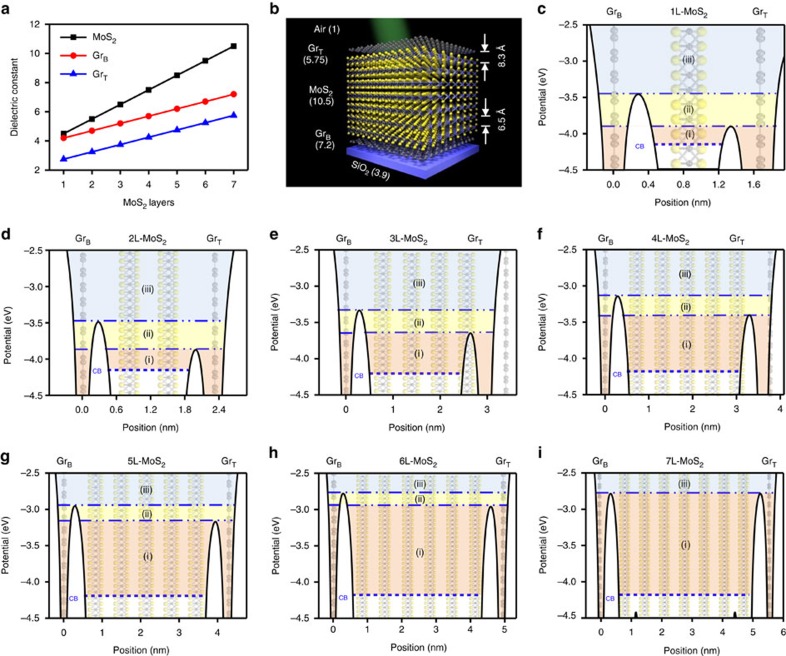
MoS_2_ layer-dependent electrostatic potentials of vdWHs. (**a**) MoS_2_ layer-dependent dielectric constant of MoS_2_, Gr_T_ and Gr_B_. (**b**–**h**) MoS_2_ layer-dependent electrostatic potentials of vdWHs. (**i**) Schematic images of graphene/7L-MoS_2_/graphene heterostructures with SiO_2_ substrate and Air environment. Dielectric constant of each layers are indicated in brackets.

The electrostatic potentials were calculated in terms of the number of MoS_2_ layers (Fig. 5). The region of fully confined between *T*_T_ and *T*_B_ case is expanded in response to the number of MoS_2_ layers due to the increased barrier heights at both sides. On the other hand, the region of partially confined at the *T*_B_ case is shrunken when the number of MoS_2_ layers increases due to the reduction of barrier height difference at both junctions. The region of no confinement case is also shrunken by increased barrier heights at both sides. Electrostatic potential is saturated over the 7L-MoS_2_ and behaves as bulk MoS_2_. The photocarrier extraction rates (*I*) were calculated in terms of the electrostatic potentials.

### Calculated photocarrier extraction rate

**Figure 6:**
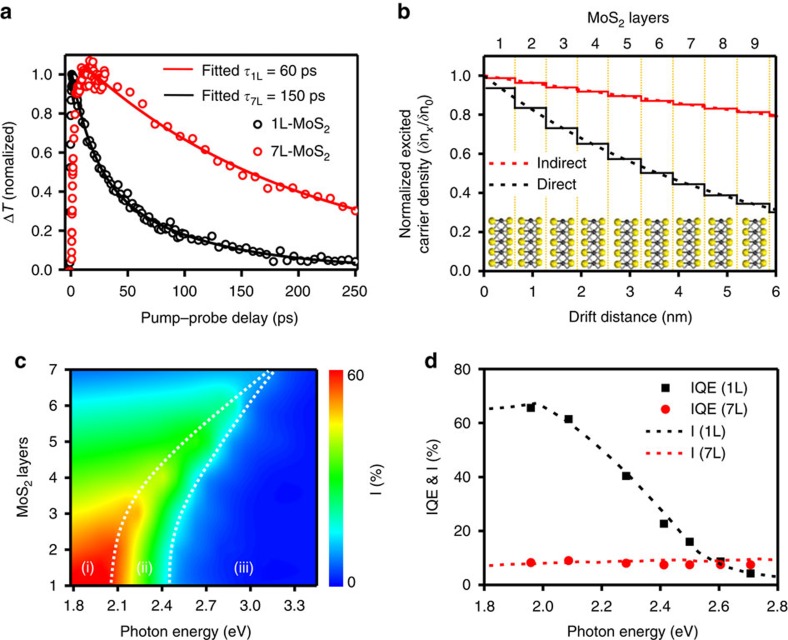
Calculated photocarrier extraction rate of vDWHs with various number of MoS_2_ layers. (**a**) Normalized dynamics of differential transmission (Δ*T*/*T*_0_) of 1L-MoS_2_ and 7L-MoS_2_ for pump fluence (dots) and fitting lines. Δ*T* is the pump-induced probe transmission change and *T*_0_ is the probe transmission without pump excitation for pump fluence. The decay lifetime of 1L-MoS_2_ (*τ*_mono_) and 7L-MoS_2_ (*τ*_multi_) obtained from the fitting. (**b**) Normalized excited electron carriers (*δn*_*x*_) by initial excited electron carriers (*δn*_0_) for direct gap MoS_2_ (black line) and indirect gap MoS_2_ (red line) along with carrier drift distance (nm). (**c**) 2D colour plot of photocarrier extraction rate as a function of the number of MoS_2_ layers and photon excitation energy. (**d**) Photon energy dependence of IQE (black dots: 1L-MoS_2_; red dots: 7L-MoS_2_) and calculated photocarrier extraction rate (black dashed line: 1L-MoS_2_; red dashed line: 7L-MoS_2_).

**Figure 7:**
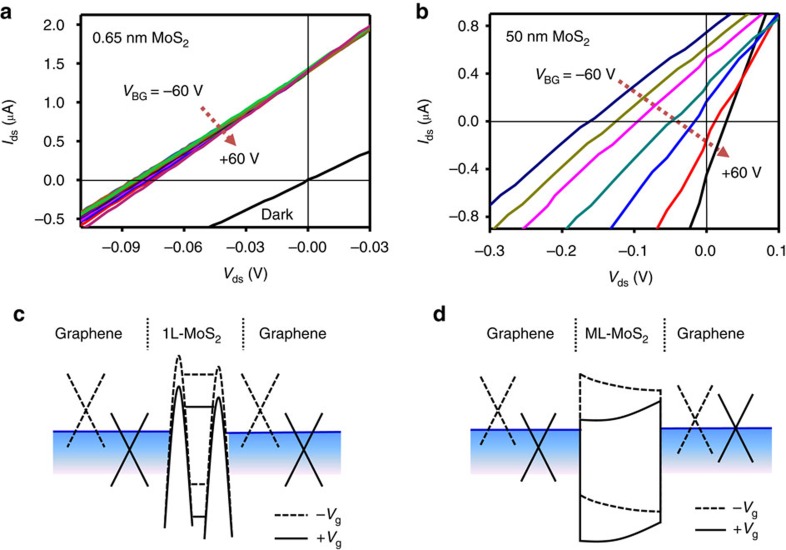
*I–V* characteristics of monolayer and ML MoS_2_. (**a**,**d**) *I*–*V* characteristics of (**a**) graphene/1L-MoS_2_/graphene and (**b**) graphene/ML MoS_2_ (50 nm)/graphene under laser illumination of 514 nm laser and dark (black line, *V*_g_=0 V). Schematic band diagram for (**c**) graphene/1L-MoS_2_/graphene and (**d**) graphene/ML MoS_2_/graphene at the negative *V*_g_ (dashed line) and positive *V*_g_ (solid line).

The carrier lifetime in direct gap (1L-MoS_2_) is shorter than that of indirect gap (7L-MoS_2_), which should be taken into account in recombination process during carrier drift. We have measured the carrier lifetime in direct gap (1L-MoS_2_) and indirect gap (7L-MoS_2_) based on transmission change in pump–probe measurement (Fig. 6a) and then calculated the normalized excited carriers (*δn*_*x*_) by initial excited carriers (*δ*_*0*_) for 1L-MoS_2_ (direct gap, red line) and ML-MoS_2_ (indirect gap, black line) along with carrier drift distance (nm; Fig. 6b and Supplementary Note 5). The normalized excited carrier density was then multiplied with photocarrier extraction rate (I) to clarify recombination difference in ML-MoS_2_. The 1L-MoS_2_ shows ultra-fast carrier transfer (∼1 ps) rate into graphene layer, much shorter than carrier lifetime in 1L-MoS_2_ (60 ps), resulting in highly efficient transfer of photo-excited carriers in MoS_2_ into graphene before recombination. Therefore, we multiplied photocarrier extraction rate (I) in 1L-MoS_2_ by 100% of normalized excited carrier density. Figure 6c clearly reveals that the photocarrier extraction rate (*I*) reaches around 65% when the number of MoS_2_ layers is reduced from 7L-MoS_2_ to 1L-MoS_2_ and the photon energy approaches the bandgap (1.82 eV). The calculated *I* are in good agreements with measured IQE of 1L-MoS_2_ (black dashed line in Fig. 6d) and 7L-MoS_2_ heterostructures (red dashed line in Fig. 6d).

### *I*–*V* characteristics

Previous studies have demonstrated an external gate voltage can be used to effectively modulate the Fermi energy of graphene and thus the potential offset and the driving force for carrier separation in graphene/MoS_2_/graphene devices^7^,^12^. We have also investigated gate voltage dependency of dark current (Supplementary Fig. 8) and photocurrent generation (Fig. 7) in the 1L-MoS_2_ and ML MoS_2_ heterostructures. Overall, the 1L-MoS_2_ device showed considerably smaller modulation of short circuit current (*I*_sc_) and open circuit voltage (*V*_oc_) by an external gate voltage (Fig. 7a) than that of ML-MoS_2_ (Fig. 7b). To understand the weak gate modulation in graphene/1L-MoS_2_/graphene device, the band diagram of the 1L-MoS_2_ and ML-MoS_2_ devices at the positive and negative gate voltage are calculated (Supplementary Fig. 9 and Supplementary Note 6) and schematically illustrated in Fig. 7c,d. It is noted that the electrostatic screening of the Gr_B_ layer is dependent on its carrier concentration, which can be varied by applied gate voltage. This factor is considered in the simulation and schematics. In general, the gate voltage can more efficiently modulate the Gr_B_ *E*_F_ than Gr_T_ *E*_F_ due to the partial screening of the gate field by the Gr_B_ layer and sandwiched MoS_2_ layers. The larger gate modulation of the *T*_B_ than *T*_T_ can create additional potential offset or band slope that leads to change in *V*_oc_. In the ML-MoS_2_ device, the gate electric field primarily modulates the Fermi level (*E*_F_) of the Gr_B_ due to the relatively strong electrostatic screening by ML-MoS_2_ (Fig. 7d), leading to a large modulation of the band slope in ML-MoS_2_ and thus a substantial modulation of the *V*_oc_ and photocurrent. In contrast for the monolayer device, the gate electric field can effectively modulate both bottom and Gr_T_ *E*_F_ because of much weaker screening effect by the ultra-thin 1L-MoS_2_ (Fig. 7c). As a result, both top and bottom potential barrier are modulated together in the graphene/1L-MoS_2_/graphene device, leading to much smaller dependence of potential offset and photocarrier extraction rate on the gate voltage.

## Discussion

In summary, we have reported an unusually efficient photocarrier generation from monolayer MoS_2_ in graphene/MoS_2_/gaphene vdWHs. Owing to intrinsically slow layer-to-layer charge transport in TMD materials, the carrier mobility in the vertical direction is usually several orders of magnitudes lower than that in lateral direction, which can seriously slow down the charge separation process in the vertical heterostructures, leading to undesired photocarrier recombination in MoS_2_ before they arrive at the current collector. Importantly, we show that this drawback can be largely overcome by using atomically thin MoS_2_ to enable an unusually high photoresponsivity up to 68 mA W^−1^ with 65% of IQE in monolayer MoS_2_. This was congruent with carrier tunnelling through asymmetric electrostatic potential barriers that exist discretely in vdWHs with monolayer MoS_2_. Conversely, the symmetric potential barrier in ML MoS_2_ reduces photocurrent via carrier tunnelling. The proposed quantum mechanical-based tunnelling mechanism provides a new theoretical framework to understand van der Waals interaction in vdWHs and to design the next generation of atomically thin optoelectronics including photodetectors and photovoltaic devices. It is noteworthy that the 2.5% EQE achieved in our graphene/1L-MoS_2_/graphene devices are comparable to the 2.4% value achieved in graphene/1L-WSe_2_/1L-MoS_2_/graphene *p*–*n* heterojonctions. Considering the absorbance in two layers stack (MoS_2_/WSe_2_) is approximately twice of that in single layer (MoS_2_), the IQE in our device is about twice higher. The lower IQE in in graphene/1L-WSe_2_/1L-MoS_2_/graphene *p*–*n* heterojunctions may be partially attributed to Shockly–Read–Hall recombination and Langevin recombination, which is not present in graphene/1L-MoS_2_/graphene devices.

## Methods

### The fabrication of the vertical heterostructures

For the fabrication of the vertical heterostructures of graphene/MoS_2_/graphene device, the graphene was grown with a chemical vapour deposition process^27^,^28^ and transferred onto Si/SiO_2_ (300 nm SiO_2_) substrate, and patterned into 8 × 30 μm strips as the bottom electrode using a photolithography and oxygen plasma etching process. The MoS_2_ flakes were then transferred onto the graphene strips through a dry transfer approach^27^. The Gr_T_ electrode was transferred and patterned on the MoS_2_ flake and Gr_B_. Directly overlapping graphene area was patterned and etched away, to avoid short circuit between the Gr_T_ and Gr_B_. The metal electrode, for probe contact or wire bonding purposes, was patterned on the bottom and Gr_T_ electrodes by e-beam lithography followed by e-beam deposition of Ti/Au (50/50 nm).

### Microscopic and electrical characterization

Electrical transport measurements were conducted with a probe station and a computer-controlled analogue-to-digital converter at room temperature. The scanning photocurrent measurements were conducted with the same electrical measurement system under a SP2 MP Film confocal microscope coupled with Ar/ArKr laser (wavelength 458, 476, 488, 496 and 514 nm) and HeNe laser (543, 596 and 633 nm). All optical measurements were carried out under ambient conditions at room temperature by using an inverted microscope coupled to a grating spectrometer with a charge-coupled device camera. The optical beams were focused on the sample with a spot diameter of ∼1 μm. MoS_2_ heterostructures were excited with a cw solid-state laser at a wavelength of 405 and 532 nm. A low laser power of ∼100 μW was used to avoid heating and PL saturation. The PL of MoS_2_ samples were calibrated by using rhodamine 6G molecules as the standard. Raman spectroscopy (RM1000 microprobe; Renishaw) was used to characterize the MoS_2_ and graphene with a wavelength of 514 nm (2.41 eV) and a Rayleigh line rejection filter. As Raman scattering efficiency has usually little layer thickness dependence, the PL spectra normalized by Raman intensity reflects directly the luminescence efficiency^16^.

### The DFT calculations

The DFT calculations were performed using the Quantum ESPRESSO code^29^. Geometry optimizations and total energy calculations are non-relativistic. Bandstructures calculated using a fully relativistic formalism are obtained using the atomic coordinates optimized in a similar non-relativistic calculation. The core electrons were described by norm-conserving, full relativistic pseudopotentials^28^ with nonlinear core-correction and spin–orbit information. The exchange correlation energy was described by the generalized gradient approximation, in the scheme proposed by Bueke *et al*.^30^. The energy cutoff was 50 Ry.

The monolayer was modelled using a supercell consisting of 4 × 4 unit cells of the monolayer material, comprising a total number of 48 atoms. The lattice parameter (*a*) used was optimized for the primitive unit cell. The supercell length along the direction perpendicular to the plane *c* is taken to be twice the lattice parameter, except for preliminary bandstructure calculations performed using a smaller *c*, which are identified in the text. The Brillouin zone was sampled using a 2 × 2 × 1 Monkhorst–Pack grid^31^.

The 7L material was modelled as a bulk crystal, using a 4 × 4 × 1 supercell. The in-plane lattice parameter was taken from the monolayer calculation and the *c*/*a* ratio was taken from experimental data^32^.

### Ultrafast optical pump–probe spectroscopy

The femtosecond laser source is based on a 250 kHz Ti:sapphire regenerative amplifier (Coherent RegA 9050), which provides a 1.55 eV photon energy, 50 fs pulse width and 6 μJ per laser pulse. The Reg A output pulse is separated into two pulses by a ratio of 7:3. The 70 % laser pulse is used to generate 3.1 eV pump–photon energy by second harmonic generation in a 1 mm-thick beta barium borate crystal. The other 30% laser beam is focused in an ultra-clean sapphire disk to generate a white-light super-continuum, which serves as a probe pulse. Both the pump and the probe pulses are focused on the 1L-MoS_2_ and 7L-MoS_2_ samples by an objective lens (Mitutoyo M Plan Apo × 10) and the delay between the two pulses is controlled by a mechanical delay stage (Newport M-IMS300PP).

### Data availability

The data that support the findings of this study are available from the corresponding author upon request.

## Additional information

**How to cite this article:** Yu, W. J. *et al*. Unusually efficient photocurrent extraction in monolayer van der Waals heterostructure by tunnelling through discretized barriers. *Nat. Commun.* **7,** 13278 doi: 10.1038/ncomms13278 (2016).

**Publisher's note:** Springer Nature remains neutral with regard to jurisdictional claims in published maps and institutional affiliations.

## Supplementary Material

Supplementary Information Supplementary Figures 1-9, Supplementary Notes 1-6 and Supplementary References
