# Supplementary material for: Unusually efficient photocurrent extraction in monolayer van der Waals heterostructure by tunnelling through discretized barriers
Source: Nat Commun. 2016 Nov 9;7:13278. doi: 10.1038/ncomms13278 (PMC5105192; doi:10.1038/ncomms13278)
Supplement: Supplementary Information — Supplementary Figures 1-9, Supplementary Notes 1-6 and Supplementary References [file ncomms13278-s1.pdf]

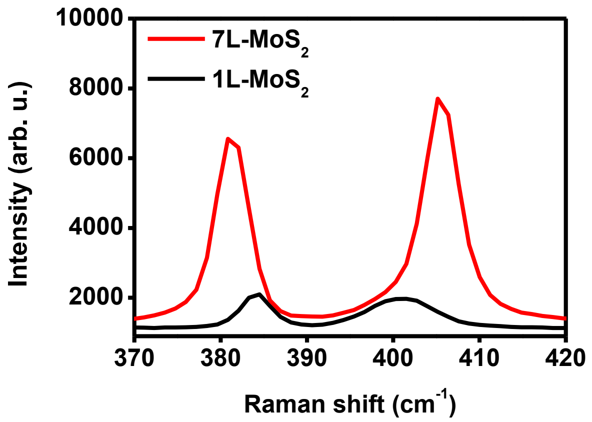

Supplementary Figure 1 Raman spectra of the Figure 1B at the 1L-MoS_2 area (black line) and 7L-MoS_2 area (red line). In the analysis of the Raman spectra of 1L-MoS_2, the peak at ~384 cm^{-1} corresponds to the in-plane (E^1_{2g}) mode, while that at ~403 cm^{-1} is attributed to the out of plane (A_{1g}) mode[1,2]. The frequency difference bsetween these two modes of 19 cm^{-1} observed in the Raman spectrum in Figure S1 can be used as a measure of 1L-MoS_2[1]. The $E^1{}_{2g}$ mode softens, and A_{1g} mode stiffens with increasing layer thickness, similar to other layered materials, where the bond distance changes with the number of layers[3]. The peak of Raman spectra of 7L-MoS_2 appeared at~381 cm^{-1} ($E^1{}_{2g}$) and~406 cm^{-1} (A_{1g}) with a frequency difference of 25 cm^{-1}. This difference can also vary as a function of doping[4].

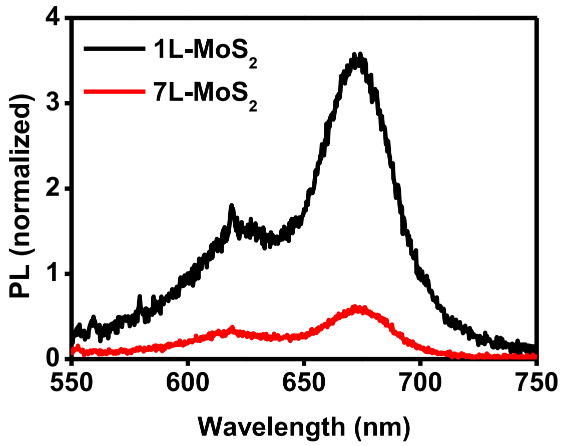

Supplementary Figure 2 Photoluminescence in 1L- (black line) and 7L-MoS_2 (red line) of the Figure 1B with illuminated wavelength of 543 nm.

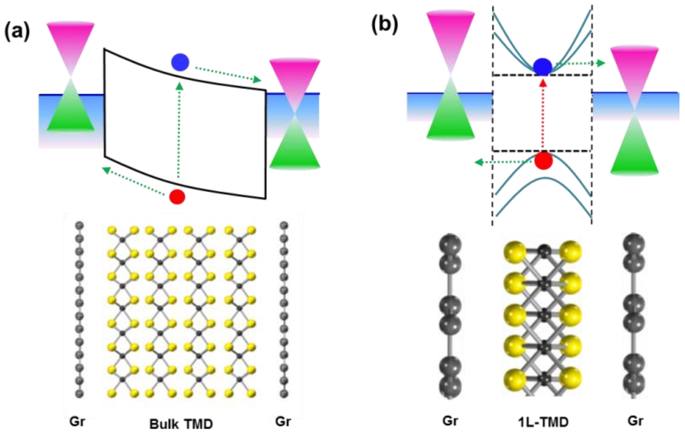

Supplementary Figure 3 Schematics of photocarrier extraction mechanisms for **a,** bulk TMD and **b,** 1L-TMD heterostructures.

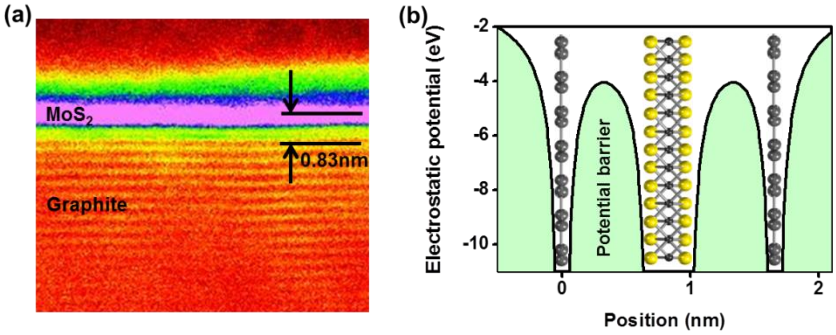

Supplementary Figure 4 a, Cross-sectional bright-field STEM image of MoS_2 on graphite. **b,** Calculated electrostatic potentials of 1L-MoS_2 heterostructures and in the vacuum environmental condition. Green colored areas indicate the potential energy barrier.

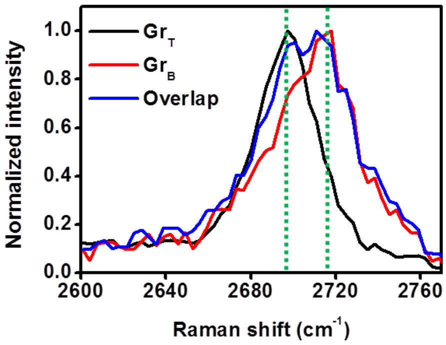

Supplementary Figure 5 Raman spectra of the top (Gr_T, black line), bottom (Gr_B, red line) graphene and overlapped area (blue line) in **Figure 1b**.

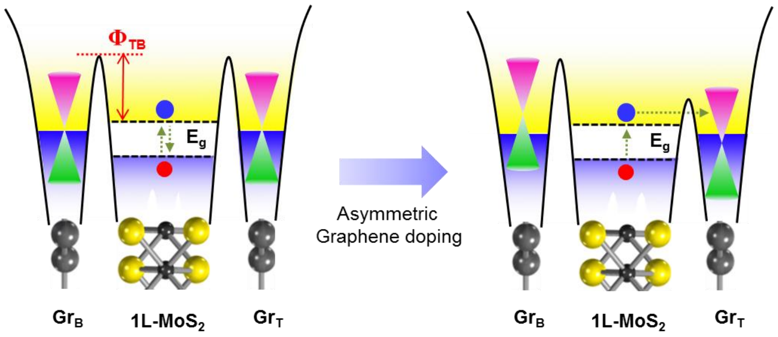

Supplementary Figure 6 Schematic images of electrostatic potentials of 1L-MoS_2 heterostructures before and after graphene doping.

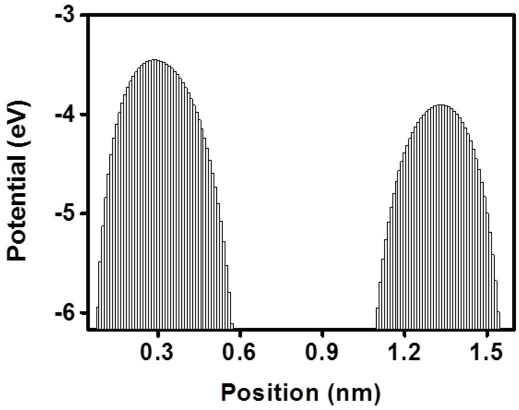

Supplementary Figure 7 Sliced square barriers with width of 0.01 nm for tunnelling probability calculation.

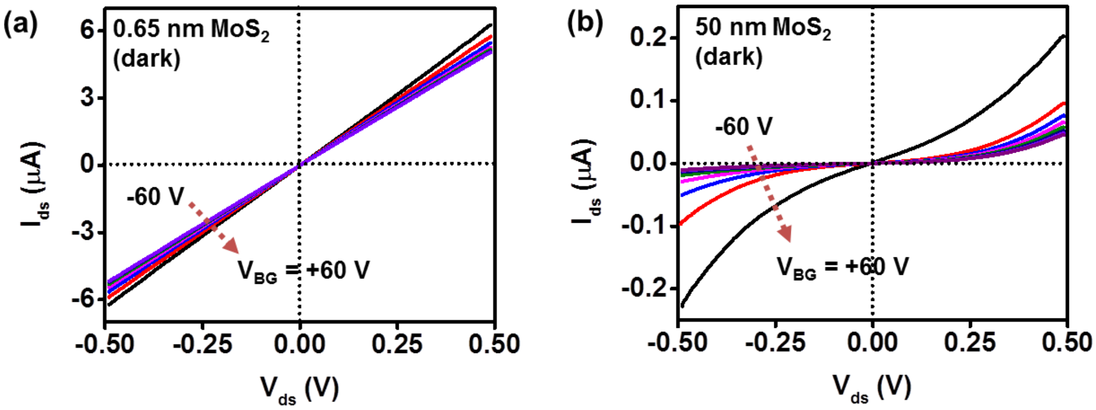

Supplementary Figure 8 a and b, I-V characteristics of (a) graphene/1L-MoS_2/graphene and (b) graphene/multi-layer MoS_2/graphene at the various V_g under dark state.

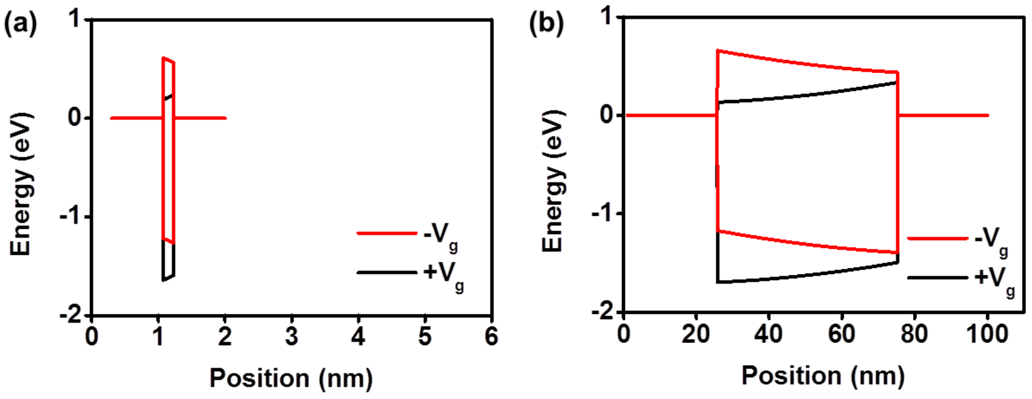

Supplementary Figure 9 a and b, Simulated band diagram of (a) graphene/1L-MoS_2/graphene and (b) graphene/multi-layer MoS_2/graphene at the negative V_g (red line) and positive V_g (black line).

Supplementary Note 1

The absorption spectra of MoS_2 was determined from the reflectance measurements[4,5]. To obtain the absorption spectrum, the reflectance spectrum from the 1L and 6L-MoS_2 flake on a glass substrate (R_{m+s}) and that from the same bare glass substrate (R_s) were measured using an optical microscope coupled with a spectrometer and CCD camera. The fractional change in the reflectance, δ_R, can be determined as the difference of these two quantities divided by the reflectance from the bare glass substrate ($\delta_R = \frac{R_{m+s} - R_s}{R_s}$). The absorbance of MoS_2 (A) can then be determined by using the relation: $\delta_R = \frac{4}{n_{sub}^2 - 1} A$, where n_{sub} is the refractive index of the glass substrate.

Supplementary Note 2

The dielectric constant of graphene is determined by the standard approximation[6,7] of $\varepsilon_{Gr} = (\varepsilon_{below} + \varepsilon_{above})/2$, where ε_{below} is the dielectric constant of the material below

graphene and ε_{avobe} is the dielectric constant of the material above graphene. If the device is in the vacuum (condition for DFT), the dielectric constant for 1L-MoS_2 heterostructure is ε_{Gr} = ($\varepsilon_{1L\text{-}MoS2}$+ ε_{vacuum})/2 = 2.7, where $\varepsilon_{1L\text{-}MoS2}$ = 4.5[8]. Figure S6b shows calculated electrostatic potential of van der Waals heterostructure device in the vacuum environment, which is highly convinced to the DFT calculated electrostatic potential (Fig. 1E).

Supplementary Note 3

2D peaks are observed at ~2700 in Gr_B and ~2716 cm^{-1} in Gr_T. The 2D band shift is the evidence of the charge doping in graphene from due to the phonon stiffening[9]. With n-/p-type doping, 2D peak of the graphene shifts in the downward/upward directions, respectively. Top graphene is more p-type doped than bottom graphene caused by different environmental reasons[10]. In the overlapping area, two distinctive peaks are observed that merged Gr_B and Gr_T peaks. It means that the doping densities in top and bottom graphenes are maintained in this overlapping area.

Supplementary Note 4

Electrostatic potential including environmental conditions is calculated by considering Coulomb interaction of top- and bottom-graphene in different dielectric constant with MoS_2 layer. In the atomic system, the electron (-charge) and nucleus (+charge, protons) are attracted to each other by Coulomb force. The Coulomb force between electron (-q) and protons (+qZ) is

$$F = \frac{(-q)(+Zq)}{4\pi\varepsilon_0 r^2} \qquad \text{[Supplementary equation 1]}$$

where r is the distance between the electron, Z is atomic number, and ε_0 is the permittivity of free space. Because the force F on a particle is equal to minus the gradient of the potential energy, the potential energy for the one electron is simply

$$E(r) = \int(-F)dr = -\frac{Zq^2}{4\pi\varepsilon_0 r} \qquad \text{[Supplementary equation 2]}$$

The excited electron is further from the nucleus than the core electrons in the valence band. In effect, the negatively charged core electrons “screen” the excited electron from the full +Ze charge of the nucleus. The excited electron would see only a net

charge of +e, and other +(Z-1)e charges are completely screened by core electrons[11]. Then the potential energy for the excited electron can be written as

$$E(r) = -\frac{q^2}{4\pi\varepsilon_0 r} \qquad \text{[Supplementary equation 3]}$$

When two nuclei are allowed to approach each other, then electron would be influenced by both nuclei according to Coulomb's law. The electron potential energy of a single electron at any point is now

$$E_p = -\frac{q^2}{4\pi\varepsilon_0 r} - \frac{q^2}{4\pi\varepsilon_0 (r+a)} \qquad \text{[Supplementary equation 4]}$$

where r is the distances between the electron and nuclei, and a is the distance between two nuclei. The electrostatic potential of graphene/1L-MoS_2/graphene can be calculated by

$$E_p = -\frac{q^2}{4\pi\varepsilon_0 r} - \frac{q^2}{4\pi\varepsilon_0 (r+0.676nm)} - \frac{q^2}{4\pi\varepsilon_0 (r+0.83nm)} - \frac{q^2}{4\pi\varepsilon_0 (r+0.984nm)} - \frac{q^2}{4\pi\varepsilon_0 (r+1.66nm)}$$

[Supplementary equation 5]

where each term is for bottom graphene, MoS_2 (sulfur, molybdenum, sulfur), and top graphene respectively. Typical distance of 0.154 nm between sulfur and molybdenum in MoS_2 was used. The distance between graphene and MoS_2 was measured to 0.83 nm by ross-sectional bright-field scanning transmission electron microscope (STEM) images (Fig. S5a).

In order to include the environmental conditions (graphene doping condition), dielectric constant of bottom graphene, MoS_2 (sulfur, molybdenum, sulfur), and top graphene can be used

$$E_p = -\frac{q^2}{4\pi\varepsilon_{GrB}\varepsilon_0 r} - \frac{q^2}{4\pi\varepsilon_{MoS2}\varepsilon_0 (r+0.676nm)} - \frac{q^2}{4\pi\varepsilon_{MoS2}\varepsilon_0 (r+0.83nm)} - \frac{q^2}{4\pi\varepsilon_{MoS2}\varepsilon_0 (r+0.984nm)} - \frac{q^2}{4\pi\varepsilon_{GrT}\varepsilon_0 (r+1.66nm)}$$

[Supplementary equation 6]

For the electrostatic potential calculation of multi-layer MoS_2 heterostructures, multiple MoS_2 layers can be simply added between bottom and top graphene layers.

In the Thomas–Fermi screening, electrons in a solid can deform the coulomb potential by electric field screening. Thomas–Fermi screening wavevector is given by

$q_{TF} = \frac{\sqrt{4\pi g_s g_v n} e^2}{k\hbar v_F}$ in 3 dimentional materials (Supplementary equation 7)

$q_{TF} = \frac{g_s g_v m e^2}{k\hbar^2}$ in 2 dimentional materials (Supplementary equation 8)

Where g_s is spin degeneracy, g_v is valley degeneracy, n is electron concentration, e is the elementary charge, k is dielectric constant, $\hbar$ is plank constant, v_F is Fermi velocity, and m is electron mass, which can deform the Coulomb potential by limits the screening length (or, equivalently, the Debye length of $\frac{1}{q_{TF}}$)[12]. The equations indicates that the Coulomb potential can be deformed depend on carrier concentration (n) in 3 dimentional materials (thick multi-layer materials), while the Coulomb potential is independent on carrier concentration (n) in 2 dimentional materials (thin few-layer materials).

Supplementary Note 5

The excited carrier continuity equation is

$$\frac{d}{dt}\delta n = D\frac{d^2}{dx^2}\delta n + \mu\xi\frac{d}{dx}\delta n - \frac{\delta n}{\tau} + g \quad \text{[Supplementary equation 9]}$$

Where δn is excited carrier density in unit volume (cm^3), D is the diffusion coefficient, μ is the mobility, ξ is the built-in internal electric field, τ is the excited carrier lifetime, and g is the generation rate of carriers[13]. In steady state, the equation is

$$\frac{d}{dt}\delta n = 0 = D\frac{d^2}{dx^2}\delta n + \mu\xi\frac{d}{dx}\delta n - \frac{\delta n}{\tau} + g \quad \text{[Supplementary equation 10]}$$

If we assume that the most of photons are adsorbed at the surface, and there is no generation of carriers in inner MoS_2, then g = 0. The equation can then be reduced to

$$0 = \frac{d^2}{dx^2}\delta n + \frac{\mu\xi}{D}\frac{d}{dx}\delta n - \frac{1}{D\tau}\delta n \quad \text{[Supplementary equation 11]}$$

General solution for this equation is

$$\delta n_x = Ae^{\frac{1}{2}\left(-\frac{\mu\xi}{D} - \sqrt{\left(\frac{\mu\xi}{D}\right)^2 + \frac{4}{D\tau}}\right)x} \quad \text{[Supplementary equation 12]}$$

where δn_x is the excited carrier density at a moving distance of x nm by diffusion and drift, and A is an unknown coefficient.

If we use δn_0 as an initial excited carrier concentration at the surface (x = 0 nm), then $\delta n_0 = A$. The excited carrier concentration at a moving distance x (δn_x) is

$$\delta n_x = \delta n_0 e^{\frac{1}{2}\left(-\frac{\mu\xi}{D}-\sqrt{\left(\frac{\mu\xi}{D}\right)^2+\frac{4}{D\tau}}\right)x}$$ [Supplementary equation 13]

The excited carriers are decayed exponentially by the recombination as the increasing the moving distance (x) increases by diffusion and drift. The diffusion coefficient (*D*) of MoS_2 to the vertical direction is obtained by Einstein relation of

$$\frac{D}{k_B T} = \frac{\mu}{q}$$ [Supplementary equation 14]

where the k_B is the boltzmann constant, *T* is the temperature, and μ is electron mobility of MoS_2 along the vertical direction.

Supplementary Note 6

The configurations of graphene/MoS_2/graphene (GMG) is considered in our simulation work. We adapt the depletion approximation, which means MoS_2 is uniformly charged, and the charge density equals its doping level. Therefore, bands of MoS_2 are parabolic instead of linear. This model is reasonable when the device channel is short, as in our cases.

For the simulation of GMG, we consider the electric field induced by gate as E_g, and then we have

$$E_g = V_g / D$$ [Supplementary equation 15]

where V_g is the gate voltage and *D* is the thickness of SiO_2 dielectric.

Then we consider electric fields in the two grapheme-MoS_2 interfaces as E_1 and E_2. The carrier density n_1 and n_2 for bottom graphene and top graphene, respectively, can be given by:

$$\varepsilon_l E_g + \varepsilon_s E_1 = n_1 e$$ [Supplementary equation 16]

$$\varepsilon_s E_2 = n_2 e$$ [Supplementary equation 17]

where ε_1 and ε_2 are the dielectric constant for SiO_2 and MoS_2, respectively.

E_1 and E_2 satisfy:

$$E_1 = E_2 + \frac{Ned}{\varepsilon_s} \qquad \text{[Supplementary equation 18]}$$

where N is the doping level in MoS_2. In our simulation, N is chosen to be 10^{17} cm^{-3}, which can be roughly estimated from transported characteristics of MoS_2 used in our study.

If we have E_1 and E_2, we can get the potential drop ΔV in MoS_2 as:

$$\Delta V = \frac{1}{2}(E_1 + E_2)d \qquad \text{[Supplementary equation 19]}$$

For graphene, we have the relation between carrier density n and chemical potential μ (Dirac point as zero) as:

$$\mu = \frac{h}{2\pi} v_F \sqrt{\pi \,|\, n - n_0 \,|} \qquad \text{[Supplementary equation 20]}$$

where h is the plank constant and v_F is the Fermi velocity, n_0 is the fixed charge graphene.

Supplementary References

1. Lee, C. *et al.* Anomalous lattice vibrations of single- and few-layer MoS_2. *ACS Nano* **4**, 2695 (2010).
2. Zhang, X. *et al.* Raman spectroscopy of shear and layer breathing modes in multilayer MoS_2. *Phys. Rev. B* **87**, 115413 (2013).
3. Arenal, R. *et al.* Raman spectroscopy of single-wall boron nitride nanotubes. *Nano Lett.* **6**, 1812 (2006).
4. Chakraborty, B. *et al.* Symmetry-dependent phonon renormalization in monolayer MoS_2 transistor. *Phys. Rev. B* **85**, 161403 (2012).
5. Williams, R. H. & Mcevoy, A. J. Photoemission studies of MoS_2. *Phys. Stat. Sol. (b)* **47**, 217 (1971).
6. Hwang, C. *et al*. Fermi velocity engineering in graphene by substrate modification. *Sci. Rep.* 5:590 (2012).

7. Elias, D. C. *et al.* Dirac cones reshaped by interaction effects in suspended graphene. *Nat. Phys.* **7**, 701 (2011).

8. Chen, X. *et al.* Probing the electron states and metal-insulator transition mechanisms in molybdenum disulphide vertical heterostructures. *Nat. Comm.* 6:6088 (2015).

9. Das, A. *et al.* Monitoring dopants by raman scattering in an electrochemically top-gated graphene Transistor. *Nat. Nanotechnol.* **3**, 210-215 (2008).

10. Britnell, L. *et al.* Field-effect tunneling transistor based on vertical graphene heterostructures. *Science* **335**, 947-950 (2012).

11. Livingston, J. D. Electronic properties of engineering materials. Wiley & Sons, Inc., New York, (1999).

12. Sarma, S. D., Adam, S., Hwang, E. H. & Rossi, E. Electronic transport in two-dimensional graphene. *Rev. Mod. Phys.* **83**, 407-470 (2011).

13. Sze, S. M. & Ng, K.K. Physics of Semiconductor Devices. Wiley, New York, (2007).
